# Floral specialization and angiosperm diversity: phenotypic divergence, fitness trade-offs and realized pollination accuracy

**DOI:** 10.1093/aobpla/plu003

**Published:** 2014-01-16

**Authors:** W. Scott Armbruster

**Affiliations:** 1 School of Biological Sciences, University of Portsmouth, Portsmouth PO1 2DY, UK; 2 Institute of Arctic Biology, University of Alaska Fairbanks, Fairbanks, AK 99775-7000, USA; 3 Department of Biology, Norwegian University of Science & Technology, Trondheim N-7491, Norway

**Keywords:** Adaptive accuracy, *Collinsia*, *Dalechampia*, fitness trade-offs, *Pedicularis*, pollination, realized precision, *Stylidium*.

## Abstract

Plant reproduction by means of flowers has long been thought to promote the success and diversification of angiosperms. It remains unclear, however, how this success has come about. Do flowers, and their capacity to have specialized functions, increase speciation rates or decrease extinction rates? Is floral specialization fundamental or incidental to the diversification? Some studies suggest that the conclusions we draw about the role of flowers in the diversification and increased phenotypic disparity (phenotypic diversity) of angiosperms depends on the system. For orchids, for example, specialized pollination may have increased speciation rates, in part because in most orchids pollen is packed in discrete units so that pollination is precise enough to contribute to reproductive isolation. In most plants, however, granular pollen results in low realized pollination precision, and thus key innovations involving flowers more likely reflect reduced extinction rates combined with opportunities for evolution of greater phenotypic disparity (phenotypic diversity) and occupation of new niches. Understanding the causes and consequences of the evolution of specialized flowers requires knowledge of both the selective regimes and the potential fitness trade-offs in using more than one pollinator functional group. The study of floral function and flowering-plant diversification remains a vibrant evolutionary field. Application of new methods, from measuring natural selection to estimating speciation rates, holds much promise for improving our understanding of the relationship between floral specialization and evolutionary success.

## Abstract (precis)

This review considers the role of reproductive factors in the evolutionary success of flowering plants, with emphasis on flowers and pollination. Flowers are complex structures that have varying degrees of integration of parts and surprising evolutionary lability. Diversification of floral form usually accompanies plant diversification by speciation. This correlation has traditionally been interpreted as the result of floral specialization increasing speciation rates. However, another possibility is that species diversity generates selection for divergent specialized flowers when related species occur together, thereby reducing extinction rates.

## Introduction

Flowering plants are the most abundant and diverse autotrophic organisms on land. Reproduction by means of flowers and fruits is often invoked as one of the main causes of this evolutionary success (Stebbins 1974; Regal 1977), but the mechanisms of the putative causal link between various angiosperm innovations and the group's evolutionary success remain elusive (Crepet and Niklas 2009). By evaluating both old and new ideas and identifying possible misconceptions, the following review attempts to come to grips with the role of pollinators, flowers and their interactions in the diversification and evolutionary success of plants.

The evolutionary success and ecological dominance of angiosperms have been associated with a number of features. Perhaps most often invoked is their use of animals to transport pollen between flowers. The capacity of extreme specialization in flower form and function is thought to have increased diversification rates (Grant 1949; Crepet and Niklas 2009) and increased the range of pollen and seed dispersal, opening new opportunities for further specialization and diversification (Stebbins 1974). These venerable ideas need further scrutiny in order to develop a programme for testing the validity of their components.

In more recent years, discussions about the types, causes and effects of floral specialization and pollination syndromes have come to the fore. At the core of many of these arguments lies uncertainty about the fundamental concept of plant fitness trade-offs, where adaptation to one type of pollinator incurs costs in terms of reduced effectiveness of another type (Aigner 2001, 2004, 2006). Is this ubiquitous, common or rare? Consensus, if there is any, seems to be that trade-offs are frequently weak or absent (see Castellanos *et al.* 2004; Muchhala 2007), although there are probably too few studies for conclusions to be drawn.

Below I address these issues by discussing pollination and floral specialization in relation to fitness trade-offs. I also review adaptive accuracy and floral precision as they relate to mechanisms of reproductive isolation, diversification and evolution of phenotypic disparity (morphological diversity).

### Evolutionary success: diversity and phenotypic disparity

Evolutionary success is commonly measured as the number of species (richness or diversity) in a clade, sometimes in combination with the ecological/morphological variation (‘phenotypic disparity’) of those species (Hunter 1998; Schluter 2000). [Phenotypic disparity is also called ‘morphological’ or ‘phenotypic diversity’, but I use ‘disparity’ here to follow a tradition already well established in the zoological literature (e.g. Wills *et al.* 1994; Foote 1997; Harmon *et al.* 2003; Hughes *et al.* 2013) and to keep a clear distinction from species diversity.] Lineages with many extant species are considered more successful than ones with very few. Lineages exhibiting greater phenotypic disparity (phenotypic diversity) are expected to be more successful because they may have greater capacity for further diversification (although the data supporting this expectation are few). The combination of relatively rapid diversification with greatly increased disparity (phenotypic diversity) is often referred to as ‘adaptive radiation’ (Simpson 1953; Schluter 2000). In turn, we can assess traits (character states) associated with evolutionary success, which are called ‘key innovations’, e.g. powered flight in insects, birds and bats, or, perhaps, flowers in flowering plants (e.g. Simpson 1944, 1953; Hodges and Arnold 1995; Hunter 1998).

**Figure 1.**
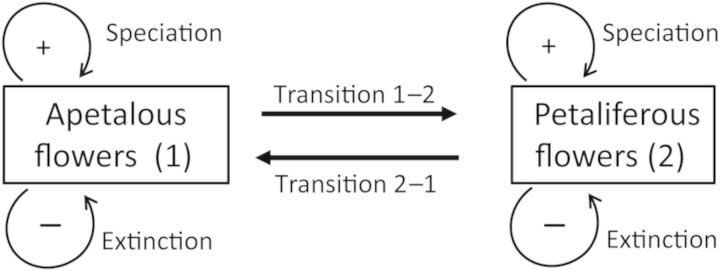
Causes of differences in species diversity of lineages with different character states, here, for example, bearing flowers with (petaliferous) and without (apetalous) petals.

There are three distinct processes that can lead to differences in evolutionary success in association with key innovations (phenotypes), the first of which is microevolutionary and the other two are essentially macroevolutionary: (i) differential trait transition rates, (ii) differential extinction rates, and (iii) differential speciation rates (Fig. 1). To illustrate the operation of these processes in more detail, consider a clade of plants where three-quarters of the species have flowers with petals, even though the basal condition is for flowers to lack petals. If petals are adaptive (say attracting more pollinators, increasing reproductive success), there may be differential transitions between states (Fig. 1), such that lineages with flowers lacking petals sometimes evolve them, but once evolved, they are rarely lost (i.e. lineages with petaliferous flowers almost never revert to being apetalous). Thus petals are a key innovation in an adaptive (microevolutionary) sense: they enhance reproductive success of individuals and populations, which in turn causes frequent microevolutionary transitions to the state. If we have enough phylogenetic information, we may recognize the pattern this process creates: parallelism (i.e. multiple origins of petals). However, multiple transitions to having petals could also be cryptic if the taxa are closely related, the phylogenetic tree is poorly resolved, taxa are missing or intermediate lineages have gone extinct. There might be only one origin detected, when actually several transitions have occurred.

Differences in the number of petaliferous and apetalous species in a clade can also come about through differential extinction. Strictly speaking this is a macroevolutionary property, for example, as when the biological ‘decks are cleared’ by astronomically induced mass extinctions (e.g. by large-meteor impacts). However, if extinction has occurred over extended periods (sometimes called the ‘background extinction’ rate), not in a single pulse, there is a likely link to normal microevolutionary processes. Thus if petals attract more pollinators, resulting in better pollination and lower rates of reproductive failure, lineages with petals would tend to experience lower probabilities of extinction.

Finally, if petals promote attraction of particular kinds of pollinators, the presence of petals might increase speciation rates by promoting pre-pollination reproductive isolation from related taxa. This could come about in three ways: (i) attracting and rewarding a set of pollinator species not used by related plant species, (ii) petals enforcing specialized handling, promoting associative learning or otherwise enhancing constancy (temporary floral specialization by individual animals), or (iii) petals causing pollen placement and stigma contact in places on the pollinator not used by other species. Verne Grant (1949, 1971, 1994*a*) classified (i) and (ii) as ethological isolation and (iii) as mechanical isolation.

### Semantic issues

#### Pollinator importance, effectiveness, efficiency and abundance

Stebbins (1970, 1974) proposed the ‘most effective pollinator principle’ which states that:

Since selection is a quantitative process, the characteristics of the flower will be molded by those pollinators that visit it most frequently and effectively in the region where it is evolving.

Although the intention of this statement is clear in this context, Stebbins' unfortunate use of ‘effective’ both in the name of the principle and as a component of importance in natural selection has led to confusion about which pollinators are expected to be driving selection. My interpretation of what Stebbins meant in this statement can be expressed by changing one word:

‘… the characteristics of the flower will be molded by those pollinators that visit it most frequently and [*efficiently*] …’

(i.e. the most *effective pollinators*). [As noted below, we should probably also insert ‘usually’ in this statement; when fitness trade-offs in pollinator use are absent, the ‘moulding’ pollinator may not be the most effective one (Aigner 2001).]

Thus I recommend we define pollinator effectiveness as the product (or a similar function) of visitation rate (frequency) and per-visit efficiency (measured as pollen delivered, seeds produced, offspring sired, etc., per visit; see also Freitas 2013). For example,

(1) $$\hskip-1pc\eqalign{\hbox{pollinator}\,\hbox{effectiveness = visitation}\,\hbox{rate} \times \hbox{efficiency}}$$

Earlier my colleagues and I have used ‘pollinator importance’ as a less ambiguous term, but conceptually related to effectiveness as defined above (Armbruster 1985, 1988, 1990; Armbruster *et al.* 2000; Fenster *et al.* 2004). We referred to the pollinator with the highest importance score as the ‘principal pollinator’. One practical estimator of pollinator importance (PI) is

(2) $$\hbox{PI} = V \times A \times S$$

where *V* is the visitation rate per unit time, *A* is the per-visit probability of contacting the anthers and *S* is the per-visit probability of contacting the stigmas (Armbruster 1985, 1988, 1990).

Freitas (2013) has come to a similar conclusion about the confusion caused by these semantics. He recommends calling visitation rate ‘visitation intensity’ and efficiency ‘efficacy’, but otherwise his recommendations largely agree with the above.

#### Floral isolation

**Figure 2.**
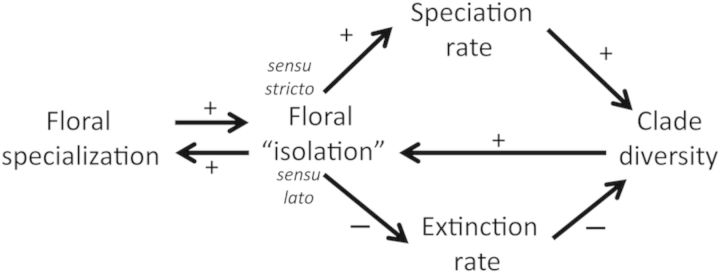
Complex links between floral specialization, floral ‘isolation’ and clade species diversity/richness. Specialized flowers may increase the likelihood of floral isolation in the strict sense and/or in the broad sense. Increased occurrence of floral isolation, in the strict sense, may increase the speciation rate and hence clade species richness and diversity. Alternatively, floral ‘isolation’, in the broad sense, may decrease the rate of extinction and lead to increases in clade species richness and thereby diversity. In turn, increased species diversity of clades may increase the number of clade members occurring in sympatry, thereby selecting for improvements in floral isolation in the broad sense, which may be manifested by increased floral specialization.

Before evaluating the role of floral specialization in diversification, it is necessary to define floral isolation precisely. Considerable confusion exists in the literature because the term has been used in at least two ways (see Fig. 2): (i) floral isolation *sensu stricto* (*s.s.*), which implies biologically significant reproductive isolation as a result of floral traits acting on their own (i.e. floral isolation as a category of reproductive isolation; see Grant 1949, 1971, 1994*a*, *b*; reviewed in Kay and Sargent 2009); (ii) floral ‘isolation’ *sensu lato* (*s.l.*), where there is some segregation of pollen flow, i.e. more intraspecific and less interspecific, without necessarily resulting in biologically significant reproductive isolation (e.g. Huang and Shi 2013; see the discussion in Kay and Sargent 2009). One problem with this dichotomy is: what is meant by ‘biologically significant’, and can it ever be defined or assessed? For the purpose of this review, I will treat ‘biologically significant isolation’ to be isolation that generates segregation of gene flow sufficient to allow, by itself, genetic divergence of the population in response to drift or weak selection. Thus narrow-sense floral isolation acting by itself generates sufficient reproductive isolation to prevent or retard genetic homogenization of populations. In contrast, broad-sense floral ‘isolation’ includes anything that reduces inter-morph mating even by a few percentage points, and may or may not have any direct genetic consequences. Because I think of floral isolation as a type of reproductive isolation and because reproductive isolation is commonly defined as ‘the inability of a species to breed successfully with related species’ (Merriam-Webster 2013), I prefer to use ‘floral isolation’ in the narrow sense. In place of ‘floral isolation *s.l.*’, I will usually refer directly to the selective mechanisms involved in reducing interspecific pollination (e.g. divergent adaptations to different pollinators, reinforcement of reproductive isolation or character displacement). Although I do not recommend abandoning the term ‘floral isolation’, I urge that we always explain in what sense we are using it.

## Floral Specialization, Fitness Trade-offs and Adaptive Accuracy

The concept of specialization has several components and meanings as applied to flowers. The manifold nature of this concept has created considerable confusion and discussion (cf. Ollerton 1996; Waser *et al.* 1996; Johnson and Steiner 2000; Fenster *et al.* 2004; Ollerton *et al.* 2007). Armbruster *et al.* (2000; see also Fenster *et al.* 2004) pointed out that specialization can refer either to a state (being specialized: ‘ecological specialization’) or to a process (becoming more specialized: ‘evolutionary specialization’). Thus, a species may be legitimately viewed as unspecialized ecologically, but specialized evolutionarily, if it is more specialized than its ancestors.

Fundamental to the definition of floral specialization is the question of what is being specialized on. Classically, specialized pollination has referred to the number of species of animals that pollinate the flowers, hence a continuous spectrum, from one species (specialized) to many species (generalized). More recently, the concept of specialization vs. generalization in pollination has focused on functional groups of pollinators. This term was apparently coined independently (but with similar meaning) by Corbet (1997—‘function group’), Johnson and Steiner (2000—‘function type’) and Fenster *et al.* (2004—‘functional group’). Pollinator functional groups are animals that generate similar selection pressures on flowers, e.g. bees with similar tongue lengths, even though they may belong to different genera or families (Johnson and Steiner 2000; Fenster *et al.* 2004). [This is similar but not the same as what Ollerton *et al.* (2007) refer to by ‘functional specialization’. ‘Functional specialization/generalization’ *sensu* Ollerton *et al.* (2007) refers instead to the number of higher taxa being pollinators (e.g. bees, birds, bats). This contrasts with what these same authors called ‘ecological specialization/generalization’: the number of *species* of pollinators. ‘Ecological specialization’ *sensu* Ollerton *et al.* (2007) differs from the use by Armbruster *et al.* (2000), Fenster *et al.* (2004) and the use adopted here.]

Ollerton *et al.* (2007) coined the term ‘phenotypic specialization’ of flowers, where the morphology, colour and/or fragrance of a flower is specialized, even though the current pollinators may not be especially restricted. Thus a phenotypically specialized flower may have ecologically generalized pollination (by multiple functional groups). This unexpected outcome may reflect a holdover from a prior history of specialization, compensatory adaptations for dealing successfully with a variety of pollinators, as may be the case for *Stylidium* spp. (Armbruster *et al.* 1994, 2004, 2009*a*, *b*; see below), or the lack of fitness trade-offs in adapting to pollinators that increase marginal fitness (Aigner 2001, 2004, 2006). Fitness trade-offs in this context are when a trait's positive effect on pollination effectiveness of one pollinator (fitness) creates a negative effect on pollination effectiveness of one or more other pollinators. Aigner (2001, 2006) explored the effect of fitness trade-offs in the evolution of phenotypic specialization, pointing out that, in the absence of steep fitness trade-offs, phenotypic specialization can evolve without restricting the number of pollinator types (ecological generalization is maintained; see the discussion in the next section). In contrast, in the presence of steep trade-offs, phenotypic specialization in response to selection by one pollinator species results in fewer functional types of pollinators that can be utilized (ecological and evolutionary specialization). Muchhala *et al.* (2010) explored the role of pollen fates in the evolution of floral specialization. Individual-based models indicated that male fitness differentials can drive the evolution of ecological and phenotypic specialization even in the absence of fitness trade-offs.

### Fitness trade-offs

The above studies underscore the importance of understanding how often fitness trade-offs between floral adaptations to different pollinators occur, not least because floral specialization is promoted by the fitness trade-offs between pollinators (e.g. Schemske and Horvitz 1984; Wilson and Thomson 1996; Aigner 2001; Mayfield *et al.* 2001; cf. Muchhala *et al.* 2010). Aigner's own studies (2001, 2004, 2006) suggested that fitness trade-offs were absent in pollination of generalist *Dudleya* flowers by large bees, small bees and hummingbirds. Aigner (2006) reviewed a number of studies that suggested trade-offs in floral adaptation to pollination by different agents, but most of these studies either failed to elucidate the functional basis of fitness trade-offs or failed to account for both components of pollinator effectiveness (abundance and efficiency), which leaves uncertainty about the importance of the trade-offs in those systems.

An elegant experimental study with *Penstemon* (Plantaginaceae) showed no detectable fitness trade-offs across traits affecting hummingbird vs. bee pollination (Castellanos *et al.* 2004). Another experimental study, which did detect clear trade-offs, is Muchhala's (2007) study of hummingbird and bat ‘pollination’. This involved artificial flowers of different widths, capturing the morphology of two species of *Burmeistera* (Campanulaceae), one primarily hummingbird pollinated (narrow floral tube) and the other primarily bat pollinated (wide floral tube). Hummingbirds were better at transferring pollen between narrow-tube ‘flowers’, and bats better at transferring pollen between wide-tubed ‘flowers’. Importantly, intermediate-width tubes (generalists) performed worse than the narrow tubes with hummingbirds and worse than the broad tubes with bats (and had lowest ‘pollination’ overall), demonstrating a clear trade-off favouring two specialized phenotypes over one intermediate generalist.

Temeles *et al.* (2009) described an intriguing system involving sexually dimorphic purple-throated carib hummingbirds (*Eulampis jugularis*) exerting selection on the shape of *Heliconia* (Heliconiaceae) flowers. Using artificial flowers, they discovered trade-offs in handling time, where females, which have long, curved bills, handled longer flowers (of all curvatures) more quickly and effectively, obtaining the deeper nectar, than the males, which have short, straight bills. In turn, males had shorter handling times than females on artificial flowers with short, straight corollas (but only when hovering). Although the measured trade-offs were experienced by the pollinator, they suggested an indirect plant fitness trade-off, where both birds and plants should specialize on the appropriate morphs, with selection against generalists (Temeles *et al.* 2013).

Schemske and Bradshaw (1999) and Bradshaw and Schemske (2003) found that variation in flower colour in hybrid *Mimulus* created a trade-off, affecting visitation rates of bees and hummingbirds in opposite directions. However, variation in nectar volume and petal surface area did not create trade-offs, having significant effects on only one of the two pollinator types (Schemske and Bradshaw 1999).

**Figure 3.**
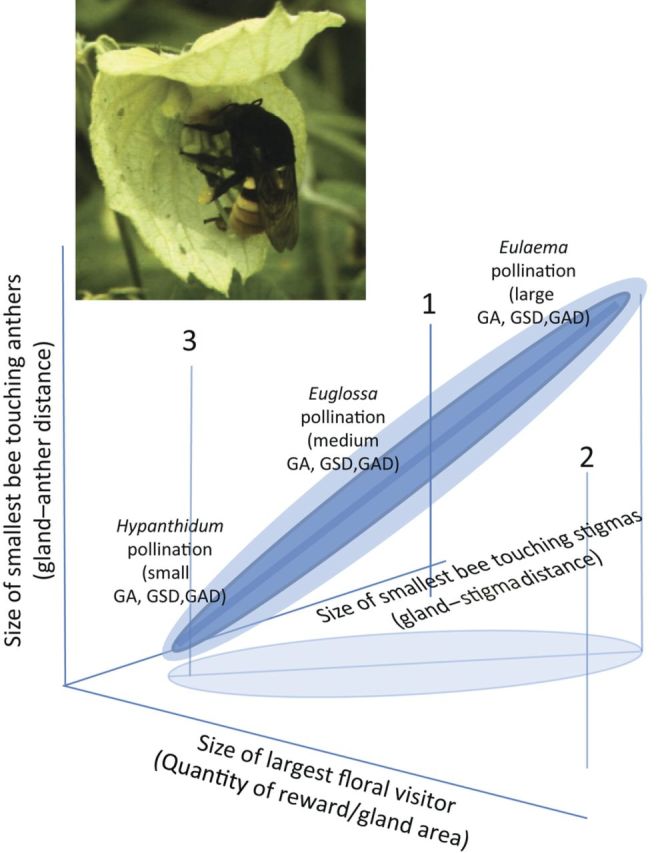
Adaptive ‘ridge’ (a series of concentric cigar-shaped volumes) in three-trait space, capturing fitness trade-offs in *Dalechampia* spp. with resin rewards. Adaptation to small-bee pollinators precludes visitation by large bees, while adaptation to large-bee pollinators precludes small-bee pollinators. The darker the shade of blue, the higher the fitness in that volume. Off-diagonal volumes (clear) experience lowest fitness. Region 1 is a volume of low fitness because only small bees are attracted, but only large bees contact stigmas and anthers. Region 2 is a volume of low fitness because resin costs exceed pollination benefits, and/or greater interspecific pollination occurs. Region 3 is a volume of low fitness because, although the small bees attracted touch the stigmas, they do not carry pollen because they do not contact the anthers.

There is also an older literature addressing fitness trade-offs in pollination using natural variation among populations and species. Trade-offs, if any, are displayed in the shapes of estimated adaptive surfaces (see reviews in Schluter 2000; Svensson and Calsbeek 2012). Because this approach provides uncontrolled comparative results rather than controlled experimental ones, it is necessary to be more cautious in interpretation, being alert to possible confounding factors. Nevertheless, clear fitness trade-offs in *Dalechampia* (Euphorbiaceae) pollination were documented using intra-population, inter-population and inter-species comparisons to estimate the shape of the adaptive surface. These plants have bilaterally symmetrical blossoms (pseudanthial inflorescences) with pollination by resin-collecting bees (which use the resin for nest construction) of a range of sizes. Analyses showed that adaptation to pollination by large resin-collecting bees (Apidae: *Eulaema*, *Eufriesea*) reduced or precluded, respectively, pollination by medium-sized resin-collecting bees (Apidae: *Euglossa*) and small resin-collecting bees (Megachilidae: *Hypanthidium*; Armbruster 1985, 1988, 1990, 2006; Hansen *et al.* 2000). This trade-off operated as an interaction between (i) attraction and visitation rate, as determined by the amount of resin reward, and (2) pollinator efficiency, measured as the product of the rates of bee contact with stigmas and with anthers. This interaction creates a positive-diagonal, adaptive ridge (in 3-trait space), which is also an axis of specialization (attraction of and pollination by small bees, medium-sized bees or large bees). Off-diagonal trait combinations have either more generalized pollination with lower fitness (attraction and pollination by all bee sizes with wasteful reward investment and loss of pollen to other sympatric *Dalechampia* species) or extremely low pollination rates (attraction of only small bees when stigmas are contacted only by large bees; Fig. 3; Armbruster 1988, 1990, 2006). All populations and species were observed to occupy the predicted adaptive ridge. This ridge is an allometric trajectory, so it is possible that the relationship is pleiotropic rather than created by a fitness trade-off, although phenotypic (Armbruster 1991) and quantitative-genetic (Hansen *et al.* 2003*a*, *b*) data suggest that this is very unlikely.

**Figure 4.**
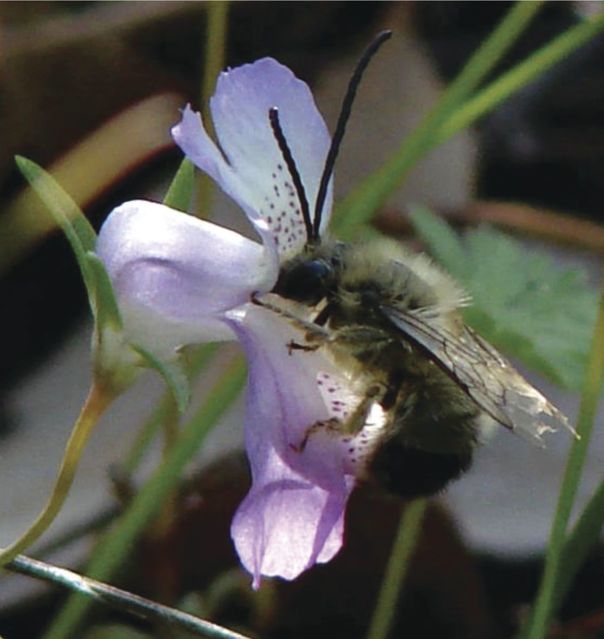
Flower of *Collinsia sparsiflora* with pollinating male *Eucera* (*Tetralonia*) bee (Apidae: Anthophorinae). The keel of the lower lip is partially depressed, with the fertile part contacting the underside of the thorax.

Another comparative study system exhibiting bilaterally symmetrical flowers and apparent fitness trade-offs is the genus *Collinsia* (Plantaginaceae). These annuals have flowers resembling pea flowers in having stamens and style enclosed in a keel-like fold of the lower corolla lobe (see Kalisz *et al.* 1999). The keel is depressed when alighting bees are of sufficient mass (in passive depression) or strength (in active depression). With depression of the keel, the stigma contacts, and/or pollen is deposited on, the underside of the bee (Fig. 4; Armbruster *et al.* 2002, 2004, 2009*a*). Variation in flower size and peduncle (floral stem) strength creates continuous variation in ‘optimal’ pollinator size. Large-flowered species attract large bees (Apidae: *Bombus*, *Anthophora*, etc.) and have strong peduncles to support the weight. These flowers are also visited by smaller bees (Megachilidae: *Osmia* spp., especially males; various Halictidae), but these bees are too small to depress the keel, and they obtain nectar (nectar thieves) or glean stray pollen without contacting anthers or stigmas. Small-flowered species of *Collinsia*, in contrast, do not usually attract large bees, both because of less nectar (Heinrich and Raven 1972) and because if large bees visit small-flowered species the peduncles collapse under their weight and the bees are dumped onto the ground (an experience the bees seem to avoid). The corolla of small flowers is lightly spring-loaded so that small bees can depress the keel and act as good pollinators. The allometry of floral size and accompanying ‘engineering’ creates an apparent trade-off, where large flowers can be pollinated by large bees but not small ones, and small flowers can be pollinated by small bees but are seldom visited by large ones. In the absence of an experiment, one cannot rule out that the apparent trade-off could be eliminated by selection for generalized pollination, but it seems unlikely.

**Figure 5.**
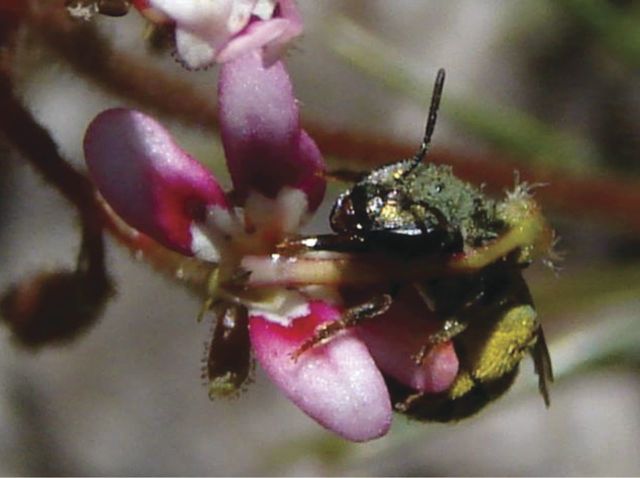
*Stylidium dichotomum* flower in female phase retrieving pollen from *Leioproctus* sp. which has sprung the column while obtaining nectar.

Perusal of published studies suggests the hypothesis that trade-offs seem more likely to occur in bilaterally symmetrical (zygomorphic) flowers than radially symmetric flowers. However, a notable exception to this trend is found in the plant genus *Stylidium* (‘triggerplants’, Stylidiaceae). These Australasian herbs and shrubs have phenotypically highly specialized, protandrous flowers that are zygomorphic and bear nectar in a tube or spur. The column (gynostemium) is formed by fused pistillate and staminate tissues and is motile, ‘explosively’ depositing pollen on, or retrieving it from, the pollinator in 10–15 ms (Fig. 5; Findlay 1978; Armbruster *et al.* 1994, 2004, 2009*a*). The flowers are ecological generalists, attracting a large number of species in several function groups of pollinators (small bees, large bees, syrphid flies, and small-, medium- and large-bodied bee flies). All functional groups are similarly effective pollinators across a range of flower sizes. The fitness contributions of pollinators are additive; there are no detectable trade-offs over the range of floral visitors in these functional groups. This is because, except for the extremes, the flowers do not experience morphological, fit-related trade-offs, where the size of the flower must match exactly the size and shape of the pollinator for pollen to be picked up and deposited, as described above. Instead the motile column behaviourally ‘adapts’ to size and shape of the diverse pollinators. Pollen is deposited and stigmas make contact in the same location on a given pollinator species. However, anther/stigma contact is in different locations on different pollinator species, but consistently so: e.g. *Stylidium lineatum* places and retrieves pollen at the tip of the abdomen of medium-sized bee flies, the top of the thorax of large bee flies and the front of the head of large anthophorid bees (W. S. Armbruster, unpubl. res.). Thus the complex, specialized *Stylidium* floral morphology and trigger behaviour work equally well with a variety of sizes and shapes of pollinators. In fact the flower could be described as phenotypically specialized to be an excellent ecological generalist.

There are good reasons also to think that most open, actinomorphic (radial) flowers (e.g. *Dudleya*) and capitulae (e.g. most Asteraceae) usually lack fitness trade-offs across a broad range of pollinator functional groups. However, this is not always the case. There are apparent trade-offs in rates of contact with stigmas for actinomorphic flowers with ‘apertures’ of some sort, where the aperture interacts with pollinator size. For example, in some *Linum* (Linaceae) and *Parnassia* (Celastraceae/Parnassiaceae) species and in most *Passiflora* (Passifloraceae), flies and bees that are too large to fit through the gap formed by petals and fertile parts are excluded from reaching nectar and are rarely good pollinators. Excessively small flies and bees have access to the nectar but usually slip past the anthers and stigmas without making contact, thus failing to pick up or deposit pollen (high visitation, low pollination efficiency; Armbruster *et al.* 2006, 2009*a*, 2014*a*; Benevides *et al.* 2013).

Ideally, all of the above comparative systems should be tested experimentally using factorial analyses with different species of pollinators and different floral phenotypes (Wilson and Thomson 1996; Muchhala 2007). However, I think it is safe to accept, at least provisionally, the results of these comparative analyses. The existence of fitness trade-offs in certain kinds of flowers, e.g. most zygomorphic, and not others, e.g. many actinomorphic, could drive evolution towards ecological specialization and perhaps greater diversification in certain groups (e.g. Sargent 2004). Indeed, phylogenetic-comparative analyses of the evolution and diversification consequences of fitness trade-offs across pollinator types should be a fruitful line of investigation.

#### Conflicting selection generated by abiotic factors and multi-species interactions with flowers

It is now well recognized that plants interact with a diversity of mutualists and antagonists, from pollinators and defending ants to nectar robbers, florivores, folivores and pathogens, in both ecological and evolutionary time (Strauss and Irwin 2004; McCall and Irwin 2006; Strauss and Whittall 2006; Irwin *et al.* 2010). For example, while advertisement traits increase the apparency of flowers to pollinators, raising fitness, they may also increase the apparency to enemies such as nectar robbers, florivores and seed predators, lowering fitness. Thus floral traits may commonly be subject to conflicting selection mediated by mutualists and antagonists, resulting in complex trade-offs. Depending on the shape of the fitness responses to the conflicting relationships, the net result may often be stabilizing selection. The role of multiple species in generating stabilizing selection may help in explaining why phenotypic selection studies that address only one interaction, as is often the case, more often document directional selection than the otherwise expected stabilizing selection.

Numerous examples of conflicting selection on floral traits have been published, and several papers review the topic thoroughly (e.g. Brody 1997; Cariveau *et al.* 2004; Strauss and Irwin 2004; McCall and Irwin 2006; Strauss and Whittall 2006; Irwin *et al.* 2010). One of the earliest well-documented examples is the observation of conflicting selection on floral fragrance and flower shape of *Polemonium viscosum* generated by ants and bumble bees in the subalpine Colorado (Galen 1999; Galen *et al.* 1987, 2011). A very recent example is the detection of conflicting selection on the size of floral bracts of *Dalechampia scandens* in Mexico, with positive selection generated by pollinating bees and negative selection generated by curculionid seed predators (Pérez-Barrales *et al.* 2013).

Additional complexity in floral evolution may result from conflicting selection generated by pollinators and abiotic factors. For example, pollinators often select for larger corolla size, but such increases exert a large cost in terms of water loss in dry environments (Galen 2000; Lambrecht and Dawson 2007). Selection to speed up the life cycle in highly seasonal environments may result in selection against large flowers, in conflict with selection generated by pollinators (Runions and Geber 2000; Mazer *et al.* 2004; Lambrecht 2013; but see also Moeller and Geber 2005).

### Adaptive accuracy of flowers

Another approach to assessing fitness trade-offs and possible connections between floral specialization and flowering-plant diversification involves adaptive accuracy theory.

This approach, which can be used in this context to quantify pollination accuracy, is also useful for assessing the ability of the flowers of sympatric species to generate segregated pollen flow and maintain reproductive isolation (or not).

Adaptive inaccuracy estimates the phenotypic load (maladaptation) that results from phenotypic departure from the optimum in a population. At the level of the population, there are at least three components, which are additive (Armbruster *et al.* 2004, 2009*a*; Hansen *et al.* 2006; Pélabon *et al.* 2012): (i) optimality of the mean, which is how far the mean of events departs from the optimum (=‘maladaptive bias’), (ii) the variance, which is how much individuals vary from the mean (=‘adaptive imprecision’), and (iii) the variance in the optimum. By extrapolation from measurement theory (Armbruster *et al.* 2004; Pélabon *et al.* 2012), these three components sum to the adaptive inaccuracy as

(3) $$\eqalign{{\rm adaptive}\,{\rm inaccuracy} = ({{\rm trait}\,{\rm mean}{\rm \ - }{\rm \ optimum}\,{\rm value}} )^{\rm 2} \cr  {\rm + trait}\ {\rm variance + optimum}\,{\rm variance}}$$

Although phenotypic selection only detects relative overall adaptive inaccuracy of flowers (as a component of reproductive fitness; Armbruster *et al.* 2004, 2009*a*, *b*), genetic response to phenotypic selection may occur through reducing the maladaptive bias (less departure from the optimum), decreasing (or increasing) the adaptive imprecision, decreasing (or increasing) the variance in the optimum or some combination of all three.

There are two new aspects of adaptive accuracy that need to be considered in the context of pollination: (i) *fundamental* accuracy and precision, and (ii) *realized* accuracy and precision (terms were derived by analogy to ecological niche concepts; see also Armbruster *et al.* 2014*b*). *Fundamental pollination accuracy* relates to measurements of optimality, precision and accuracy taken from the flower itself. This is only a predictor of the actual accuracy in play ecologically, the *realized pollination accuracy*. The latter then refers to the interaction of floral parts with the pollinator itself. It reflects the effects of the pollinators' behaviour in interacting with the flower (e.g. variation in approach and landing), the distribution and redistribution of pollen on the pollinator and effects of other ecological factors (e.g. other pollinators, florivores, predators). The realized precision of pollination is nearly always lower than the fundamental precision, and therefore this asymmetry also holds for accuracies.

## Mechanisms of Diversification

From the overview presented in the Introduction, it is clear that flowers may influence diversification in several ways, involving both adaptive (trait transitions, reduced extinction rates, adaptive/ecological speciation) and non-adaptive processes (e.g. non-adaptive speciation). Because the influence of adaptive processes on ‘ecological’ and ‘adaptive’ speciation has been reviewed recently elsewhere (Dieckmann *et al.* 2004; Johnson 2006, 2010; Nosil 2012; van der Niet and Johnson 2012), it is not covered in detail here. However, it should be remembered that the effect on plant speciation of adaptation to pollinators is potentially of great importance (Johnson 2006, 2010; van der Niet and Johnson 2012). In both allopatry and sympatry, adaptive divergence of floral traits can lead indirectly to the establishment of barriers to interbreeding. Additionally, adaptive reinforcement of reproductive isolation (‘Wallace effect’; Grant 1966; Silvertown *et al.* 2005) and reproductive character displacement (Grant 1972) may further promote speciation rates and hence diversification. In contrast, non-adaptive speciation, even if rare, is of conceptual importance because it potentially decouples species-level selection (Stanley 1975) from microevolution (Gould 2002). The commonest form of non-adaptive speciation (in the sense of the biological-species definition) in plants is probably polyploidy. Non-adaptive (‘instantaneous’) speciation has also been suggested for the origins of fragrance ‘races’ and new species in male-euglossine-pollinated orchids and sexually deceptive orchids (Dressler 1968; Cozzolino and Widmer 2005).

### Specialization and clade success

Features that promote specialization are commonly interpreted as key innovations, although there are reasons to be cautious in making this assumption. For example, dependence on a single obligate mutualist may greatly increase the risk of extinction (Waser *et al.* 1996). Nevertheless, the relationship between specialization and clade ‘success’ has fascinated biologists since Darwin. Botanists and zoologists have often thought about the relationship between these two properties somewhat differently. Zoologists have largely considered specialization to be adaptive, leading to adaptive evolution and evolutionary success (higher transition rates and lower extinction rates; e.g. Schmidt-Kittler 2002; Fernández-Marín *et al.* 2004; Litman *et al.* 2011; Eastman *et al.* 2013). Surprisingly, evolutionary botanists, in contrast, have probably written more, at least implicitly in early literature, about non-adaptive processes such as non-adaptive speciation, where, for example, use of different pollinators is thought to lead incidentally to reproductive isolation and thereby increase speciation rates (Grant 1949, 1971, 1994*a*; Hodges and Arnold 1995; Hodges 1997; Sargent 2004; but see Stebbins 1974; Johnson 2006). (Ecological speciation is included in this argument, but it is less clear how adaptive specialization on different pollinators affects speciation rates.)

The role of floral specialization in reducing extinction rates is rarely discussed, although Hodges and Arnold (1995) mention ‘increased reproductive success’ as a possible outcome of floral specialization. Research on the role of plant physiology and leaf and stem anatomy and function in evolutionary success also emphasizes the adaptive nature of specialization (and implicitly reduced extinction; Gianoli 2004; Klak *et al.* 2004; Bakker *et al.* 2005; Donoghue 2005; Guzman *et al.* 2009; Givnish 2010). Although Johnson and colleagues (Johnson 2006, 2010; van der Niet *et al.* 2006; van der Niet and Johnson 2012) have emphasized the role of adaptation to pollinators in both allopatric and sympatric divergence, it is largely in the context of speciation rather than reproductive success or population viability.

It seems clear that floral specialization is often associated with clade success (species richness; Hodges and Arnold 1995; Hodges 1997; Sargent 2004; Kay and Sargent 2009). However, it is less clear what causes this association (see below; Armbruster and Muchhala 2009).

### Reduced extinction

As already noted, it is commonly suggested that flowering plants are successful and species rich because reproduction by means of flowers and fruits is more effective overall than by means of strobili (e.g. Stebbins 1974). Indeed, typical angiosperm flowers provide numerous potential advantages over gymnosperm strobili. These include more rapid development, which may have been important in colonizing and reproducing in comparatively short-lived or highly seasonal habitats. Flowers are usually smaller than ovulate strobili, which allows greater flexibility in reproductive investment. Additionally, most flowers are hermaphroditic, leading to efficiencies in pollination by animals, as well as potential reproductive assurance by autonomous self-pollination in the event of pollinator failure. Another innovation is that flowers provide an arena for extended pollen-tube competition, which may promote offspring fitness by choice of superior sporophytic fathers (Mulcahy 1979; Skogsmyr and Lankinen 2002; Pannell and Labouche 2013). Pollen competition may also screen out genetically inferior male gametophytes, which potentially reduces inbreeding depression and might promote the persistence of mixed mating systems (Armbruster and Rogers 2004; Goodwillie *et al.* 2005; Lankinen and Armbruster 2007; cf. Igic and Busch 2013). The structures and secretions of angiosperm flowers also promote using animals for pollen and seed dispersal; this may have allowed populations to be more dispersed and achieve targeted colonization of favourable microenvironments (Stebbins 1974; Regal 1977; Crepet and Niklas 2009). All of these features should lead to greater population persistence and hence lower extinction rates.

Another advantage of angiosperm flowers is that they are modular units comprising many parts of differing degrees of integration. As modules, they can maintain some degree of phenotypic independence from variation in the rest of the plant, which is adaptive in the face of pollinator-mediated stabilizing selection for flower size and shape (Berg 1960; Armbruster *et al.* 1999, 2004; Hansen *et al.* 2007; Pélabon *et al.* 2011; Murren 2012). Floral parts themselves range from nearly independent to highly integrated statistically (Conner and Via 1993; Conner and Sterling 1995, 1996; Armbruster *et al.* 1999, 2004; Herrera 2001; Herrera *et al.* 2002; Anderson and Busch 2006; Hansen *et al.* 2007; Ordano *et al.* 2008; Pélabon *et al.* 2011; Alcantara *et al.* 2013) or structurally/developmentally (e.g. Armbruster *et al.* 1994, 2004). This variation in degree of integration allows tremendous adaptability in response to selection by different pollinators. For example, if sepals are under different selection than petals, their quasi-independence allows different evolutionary responses (e.g. Armbruster *et al.* 1999). If styles and stamens, for example, are selected to be of the same length (integration, in the face of variation), they can do so even though other floral traits experience different selective pressures (Conner and Via 1993; Conner and Sterling 1995, 1996). Numerous semi-independent floral parts also allow the evolution of a diversity of complex interactions with pollinators, and, in fruit, seed dispersers (Stebbins 1974).

The above arguments imply reduced extinction rates for flowering plants compared with gymnosperms. The rapidity of floral development presumably also enabled them to occupy new environments, such as highly seasonal and successional habitats (including post-fire sites) requiring rapid growth and quick reproduction. Such habitats were thought to have become more abundant during the early and mid-Cretaceous, when much angiosperm evolution was occurring (Axelrod 1970; Raven and Axelrod 1974; Stebbins 1974; Lamont and He 2012). Flower structure and complexity as described above suggest further that flowering plants had the potential to radiate by use of different pollinators and seed dispersers (see Evolution of floral disparity, below).

The above features lead to potentially rapid and labile evolutionary response of flowers to divergent selective pressures. This allows for increasing floral disparity between species in sympatry and species in allopatry but later in secondary sympatry. This capacity to diverge and specialize may thus also reduce extinction rates because it allows the compatible packing of more species (with narrower pollination niches) into communities (i.e. with minimal reproductive costs incurred by sharing pollinators).

### Increased speciation rates

A classical explanation for high angiosperm diversity is that specialized flower–pollinator relationships increase speciation rates. Thus, in addition to reducing extinction rates and increasing the opportunity for the evolution of disparity (see below), flowers with the right combination of traits may have contributed to increased speciation rates in the angiosperms. Arguments and evidence for this relationship come from three sources: (i) classical floral-isolation models, (ii) phylogenetic-comparative analyses, and (iii) ecological genetic experiments.

#### Classical floral isolation model

As noted above, Grant (1949, 1971, 1994*a*) recognized the potential importance of certain kinds of flowers in increasing speciation rates through establishment of pre-zygotic isolating mechanisms, specifically through differences in pollination ecology (floral isolation *ss.*). Complex flowers may attract only certain species of pollinators, which could lead to floral isolation from related species attracting other pollinator species. This form of ethological isolation is based on traditional ideas of specialization in flower–pollinator relationships, as captured in older syndrome literature (see Faegri and van der Pijl 1979). Some relationships between plants and their pollinators are sufficiently specialized that ethological isolation may influence speciation and reinforcement (e.g. in sexually deceptive orchids and plants pollinated by male-euglossine bees; Dressler 1968; Cozzolino and Widmer 2005; Pansarin and do Amaral 2009; but see Armbruster *et al.* 1992), but this is almost certainly the exception not the rule in angiosperms (Waser 1998, 2001; Armbruster and Muchhala 2009).

Some of the most intriguing data on ethological isolation in orchids come from comparisons of food-deceptive and sexually deceptive orchids (Scopece *et al.* 2007; Cozzolino and Scopece 2008). Generally, sexually deceptive orchids have strong pre-mating isolation and weak post-mating barriers, while food-deceptive orchids have strong post-mating isolation and weak pre-mating barriers. Sexual deception reflects unusual specialization based on chemical exploitation of one or a few pollinator species, whereas pollination in food-deceptive orchids is less specialized, attracting a range of pollinators much as do food-reward flowers. Interestingly, post-mating isolation has evolved in a clock-like manner, whereas pre-mating barriers have not, supporting the idea that, for angiosperms with food-deception and food-reward pollination systems, speciation commonly occurs through gradual divergence in allopatry (Cozzolino *et al.* 2005; Cozzolino and Scopece 2008).

Another form of ethological isolation operates through flower constancy, the tendency of individuals of some species of animals to be temporarily faithful to a single flower species or morph (Grant 1950; Waser 1986). There are several possible reasons that animals (mostly bees) may adopt constancy as a foraging strategy, but the consensus is that it is a way to reduce handling time and possibly search efficiency. It can be expected that floral features that increase a bee's learning time for handling will promote greater constancy, although supporting data are sparse and weak (Chittka *et al.* 1999; Armbruster *et al.* 2014*b*). If this is the case, however, it could result in higher speciation rates in complex flowers and hence explain greater clade diversity (e.g. Sargent 2004; Kay and Sargent 2009). However, constancy is unlikely to be a sole mechanism of reproductive isolation between incipient species in sympatry. This is because it is rare for any animal to be perfectly constant. For example, bumble bees, which are often highly constant, still make occasional (to numerous) interspecific-flower transitions (Heinrich 1976). Euglossine bees can be inconstant when flower handling is similar across plant species (e.g. Armbruster and Herzig 1984). Indeed Chittka *et al.* (1999) state: ‘…there is good evidence against the notion that pollinator constancy is involved in speciation or maintenance of plant species integrity’. Nevertheless, there are reasons to expect that plants will have evolved constancy-promoting floral features, because these increase reproductive fitness (dispersing more pollen to conspecific stigma and receiving more conspecific pollen), even though these rarely play a role in reproductive isolation. Instead, because floral features increasing constancy probably play a role in enhancing reproductive success, they may have increased diversification rates by reducing extinction rates (see above).

Certain floral traits may increase speciation rates through enhancement of mechanical isolation (Grant 1949, 1971, 1994*a*), where pollinators are restricted to a subset of visitors by the specialized fit of flowers to ‘preferred’ pollinators, or where flowers place pollen in a specific place on the pollinator (with stigma contact in the same location) not used by other species. Early studies of orchid speciation illustrate this concept nicely. As noted above, if two male-euglossine pollinated orchids produce chemically different fragrances, they are ethologically isolated (different bees attracted; Dressler 1968; Pansarin and do Amaral 2009). When visitors overlap, however, often related species diverge in the size of the flower and the size of the effective pollinator (mechanical isolation; Dodson 1962; Dressler 1968). In other cases, related orchids share visitors but partition where on the pollinator pollinaria are placed (mechanical isolation; Dressler 1968).

#### Phylogenetic evidence

Under the assumption that greater clade species richness reflects higher speciation rates, several authors have used phylogenetic data to suggest that animal pollination increases speciation rates over abiotic pollination (Dodd *et al.* 1999) or that plants with more specialized pollination have higher speciation rates than those with less specialized pollination (Hodges and Arnold 1995; Hodges 1997; Sargent 2004; Kay and Sargent 2009; Schiestl and Schlueter 2009). Of course, most of these authors acknowledge that lower extinction rates could also have played a role in this association (Armbruster and Muchhala 2009; Kay and Sargent 2009).

#### Ecological genetic experiments

A series of elegant experiments with two *Mimulus* species (Phrymaceae), *M. cardinalis* and *M. lewisii*, has shown the genetic basis of floral signals determining flower choice by pollinators (Schemske and Bradshaw 1999; Bradshaw and Schemske 2003; Ramsey *et al.* 2003). This approach is very powerful, although it was not possible to show complete isolation being generated by the detected genetic differences (see Waser 2001). Similar studies identifying the genetic basis of differences in pollinator attraction between related plant species have now been conducted in several other lineages, e.g. *Ipomopsis* (Polemoniaceae; Nakazato *et al.* 2013), *Iris* (Iridaceae; Brothers *et al.* 2013), and *Petunia* (Solanaceae; Hermann *et al.* 2013). However, it remains a significant challenge to distinguish between the role of floral signals in generating or maintaining reproductive isolation vs. simply improving reproductive fitness in sympatry. This challenge plagues all the approaches reviewed here.

#### Problems with the enhanced speciation model

It seems clear that floral specialization is often associated with clade success (i.e. species richness; Hodges and Arnold 1995; Hodges 1997; Sargent 2004; Kay and Sargent 2009). However, it is less clear what causes this association. Armbruster and Muchhala (2009) laid out several possible causes of the association between floral specialization and clade species richness (see Fig. 2), and suggested several lines of evidence that could be used to distinguish between them. Data to date are sparse, although circumstantial cases have been made. Given that only a tiny amount of inter-morph pollination will swamp any divergence except that driven by very strong selection (Wright 1951; Roughgarden 1979; Waser 2001; Armbruster and Muchhala 2009), it is important to assess the degree of reproductive isolation that can result from different kinds of floral specialization. It is not clear that the fidelity of pollinator species and individuals visiting specialized flowers (ethological isolation; cf. Waser 1998, 2001; Chittka *et al.* 1999) is great enough to preclude the minimal pollen flow needed to swamp divergence. It seems even less likely that pollen placement and stigma contact locations usually differ between related species (mechanical isolation; Grant 1994*a*) sufficiently to preclude inter-morph pollinations swamping genetic divergence (Waser 2001; Armbruster *et al.* 2014*b*; Armbruster and Muchhala 2009).

Waser (1998, 2001) pointed out that most flowers (at least in the North Temperate Zone; cf. Johnson and Steiner 2000; Armbruster 2006) have fairly generalized pollination and are visited by several to many pollinator species. It would therefore be difficult for ethological isolation at the pollinator-species level to be complete enough to provide more than a limited degree of assortative mating. Thus, ethological isolation may often enhance reproductive fitness in sympatry, but it is unlikely to maintain species ‘integrity’. Although Kay and Sargent (2009) and many others have suggested it acts multiplicatively along with other isolating mechanisms to generate complete reproductive isolation, the effect of differential attraction of pollinators alone seems too weak to be responsible for raising speciation rates in most cases (euglossine-pollinated and sexually deceptive orchids discussed above are possible exceptions).

## Evolution of Phenotypic Disparity

As noted above, phenotypic disparity refers to the phenotypic variation exhibited among related species (also called ‘morphological’ or ‘phenotypic diversity’; Foote 1997). Speciation is usually associated with disparity, but clades vary dramatically in terms of how much disparity is generated with speciation. Pollinator shifts can be viewed as ecological disparity (a component of phenotypic disparity), which may or may not be associated with morphological disparity or speciation. The evolutionary timing of disparity is something that has received attention in the literature on animals and fossils but has not been addressed, as far as I know, in the phylogenetic-comparative literature on plant species. The several studies of animals suggest that the rate of increase in phenotypic disparity rises early in most adaptive radiations and then declines (e.g. Lumbsch *et al.* 2010; Hughes *et al.* 2013). It remains to be established whether this is a general trend or if it is true for flower traits.

### Pollinator-mediated divergence and pollinator shifts

The diversity of extant pollinators and associated floral phenotypes, as reflected in pollination syndromes, attests to the importance of pollinator-mediated adaptive divergence of flowering plants (Fenster *et al.* 2004). However, the level and mechanisms by which this divergence occurs cannot be readily inferred from such broad flower–pollinator associations. Most insights into the possible processes of floral divergence come from studies of pollination ecotypes or of closely related species (e.g. congeners) in a phylogenetic context.

Wilson and Thomson (1996) recognized five processes potentially increasing floral disparity (divergence): (i) adaptation to distinct pollination niches, (ii) character displacement, (iii) adaptive wandering (where temporal variation in selective pressures can result in divergence without an overall difference in net selection), (iv) character correlations (where selection on one trait causes correlated response in another), and (v) genetic drift. These processes are detected primarily by comparing populations or species, which I review briefly below.

Armbruster and colleagues (Armbruster 1993; Armbruster and Muchhala 2009) have tried to classify and quantify the types of shifts in pollination systems. We recognized three types of shifts: (i) quantitative shifts, where transitions occur through small cumulative changes in quantitative traits in response to selection mediated by quantitative shifts in importance of different pollinators; (ii) qualitative shifts (e.g. colour, reward chemistry) with an intermediate phase when both old and new pollinators are present and effective; and (iii) qualitative shifts without an intermediate phase, where a qualitative change in floral features results immediately in a new pollinator. The first and second shifts are consistent with Stebbins' (1970, 1974) ‘gradualistic principle’, whereas the third is consistent with instantaneous speciation (see Divergence of species). While some detailed genetic studies appear to support the instantaneous pollinator-shift model (Schemske and Bradshaw 1999; Bradshaw and Schemske 2003), more recent genetic studies suggest incremental multi-locus change, supporting the gradual-divergence model (Dell'Olivo and Kuhlemeier 2013; Nakazato *et al.* 2013). Below I review additional evidence for, and criticisms of, these models.

### Divergence of populations

Comparing the pollination biology of conspecific populations can reveal divergent selective pressures, the origins of pollination ecotypes and character displacement.

#### Divergent selective pressures

Galen and collaborators were among the first to show local variation in phenotypic selection apparently generating pollination ecotypes; divergent selection was associated with genetic and floral-morphological differentiation between *Polemonium* (Polemoniaceae) populations in the alpine and subalpine (Galen and Newport 1988; Galen 1989; Galen *et al.* 1987, 1991). A similar, recent study also found differences in selection and floral morphology in alpine and subalpine *Trollius* (Ranunculaceae; Zhao and Huang 2013). Subsequent to the landmark study by Galen and her collaborators, a number of additional studies have documented spatial variation in phenotypic selection gradients; however, very few have documented differences in selection gradients consistent with patterns of phenotypic divergence (see the discussion in Maad *et al.* 2013). In the absence of this information, it is difficult to ascertain if spatial variation in selection gradients detected in any one study year actually represents long-term spatial differences in selection, given the extent of year-to-year variation in selection gradients (e.g. Schemske and Horvitz 1989; Parra-Tabla and Vargas 2004; Rees and Ellner 2009; Morales *et al.* 2010; see Siepielski *et al.* 2009). One way to increase confidence in detecting evolutionarily meaningful divergent selection is to modify potential selection experimentally, as Sletvold *et al.* (2013) have done by manipulating both pollination and the height of the graminoid/herbaceous vegetation surrounding the orchid flowers under study; they showed that variation in the height of surrounding vegetation can generate significant variation in pollinator-mediated selection on floral traits. Ehrlen *et al.* (2002) and Sletvold *et al.* (2010) provide examples of another important experimental manipulation in floral phenotypic-selection studies: comparing plants subjected to both manual and natural-pollination treatments to identify phenotypic selection mediated by pollinators.

#### Pollination ecotypes

Ecotypic divergence results from genetic response to divergent selection on conspecific populations that are geographically separated (usually reproductively isolated by distance). Pollination ecotypes are conspecific populations of plants that have diverged in pollination ecology, where that divergence has a genetic basis. (For examples of pollination ecotypes in addition to those discussed below, see the special issue of *Annals of Botany* 113(2) (2014).) Selective drivers of this divergence may be direct differences in abundance, reliability or behaviour of different pollinator species, or indirect differences in pollinator availability imposed by competing flower species. Genetic divergence across plant populations is reflected in differences in pollination ecology, including (i) divergence in pollinator species attracted as a result of different floral morphology and/or chemistry (rewards, advertisement colour or fragrance), (ii) divergence in which subset of floral visitors is used as pollinators, often as a result of differences in flower size, (iii) divergence in where anthers and stigmas contact the pollinator (without divergence in pollinator species), and/or (iv) divergence in time of day or season that flowers are receptive for pollination.

Most studies of pollination ecotypes have considered divergence in the pollinator species attracted. For example, Breedlove (1969) showed that a species of *Fuchsia* (Onagraceae) in Chiapas, Mexico, attracted and was pollinated by both bees and hummingbirds in most sites, but where sympatric with another species of *Fuchsia*, the first species specialized on attracting hummingbirds. This is an example not only of ecotypic differentiation but also of character displacement. Whalen (1978) showed character displacement and ecotypic differentiation in flower size and pollinators and in populations of Mexican *Solanum* (Solanaceae). Miller (1981) documented among-population variation in flower colour and nectar-spur length in *Aquilegia caerulea*; the variation appeared to be associated with differences in pollinating hawk-moth faunas. Inoue (1983) described pollination ecotypes in *Platanthera* (Orchidaceae) in Japan. Armbruster (1985) found a similar pattern of differential attraction of pollinators of different sizes to populations of *Dalechampia scandens* producing different amounts of reward. Robertson and Wyatt (1990) found that populations of *Plantanthera ciliaris* (Orchidaceae) in the Appalachian Mountains and coastal plain of the southeastern USA were pollinated by different *Papilio* (swallowtail) species, and these had different proboscis lengths; the orchid populations (ecotypes) diverged in nectar-spur length in the direction that matched the difference in pollinator proboscis lengths. Galen *et al.* (1991) documented ecotypic divergence in floral morphology and volatile chemistry in *P. viscosum* in the Rocky Mountains, resulting in attraction of bumble-bee pollinators in alpine populations and fly pollinators in subalpine populations.

Since these early studies of pollination ecotypes attracting different pollinators, the field has grown markedly. Johnson, Anderson and collaborators have documented pollination ecotypes involving phenotypic divergence and attraction of different pollinators to conspecific populations of *Gladiolus* (Iridaceae) and several orchids in South Africa (e.g. Johnson 1997, 2010; Johnson and Steiner 1997; Anderson *et al.* 2010; Peter and Johnson 2014). Valiente-Banuet *et al.* (2004) found that populations of *Pachycereus* cacti in tropical Mexico specialized on resident bat pollinators (flowers open and nectar secretion only at night), whereas more northerly subtropical populations had more generalized pollination involving diurnal insects, probably birds and migratory bats (flowers open and nectar secretion both during day and night). Arroyo, Pérez-Barrales and collaborators found that geographic variation in floral traits and phenotypic integration of *Narcissus papyraceus* flowers was associated with differences in pollinating faunas (Pérez-Barrales *et al.* 2007; Santos-Gally *et al.* 2013). In a classical reciprocal-transplants study of two *Platanthera* orchid ecotypes in Sweden, Boberg *et al.* (2014) showed that the long-spurred forest ecotype had higher reproductive success in the forest environment than did the meadow ecotype (although the reverse transplant experiment did not detect any difference), consistent with the differences in proboscis length of the main pollinators, and as expected from Darwin's hypothesis of spur-proboscis-length coevolution. Finally, clinal ecotypic variation in floral morphology and associated pollinator faunas have been reported for South American *Nicotiana* (Solanaceae) in Argentina (Nattero *et al.* 2011) and *Campanula* (Campanulaceae) in Norway (Maad *et al.* 2013).

Another axis of ecotypic divergence is using different subsets of visitors as pollinators. For example in *Dalechampia*, large resin-gland-to-stigma distances preclude pollination by small resin-collecting bees, even though they may visit and obtain rewards (Armbruster 1988). *Dalechampia scandens* ecotypes vary in gland–stigma distances, so some ecotypes utilize small bees as pollinators and others utilize only large bees (Armbruster 1985). A similar example is that of euglossine-pollinated orchids employing only a subset of the bees attracted as pollinators (Dodson 1962; Dressler 1968), although the observed differences here occur at the level of closely related species. Ecotypic or subspecies variation in flower size in plants is common (e.g. Baldwin *et al.* 2011; Pettengill and Moeller 2012; Maad *et al.* 2013), and in many cases this probably reflects similar patterns of divergence in utilization of pollinators; however, the drivers of such differentiation remain to be described in this context, being more often attributed to mating-system differences (e.g. Armbruster *et al.* 2002; Elle and Carney 2003; Pettengill and Moeller 2012).

A third axis of ecotypic divergence is in the location of pollen placement on, and stigma contact with, pollinators. Grant (1994*a*, *b*) discussed this in the context of interspecific reproductive isolation, and it can be expected to be common at the level of ecotypes. However, there are only a few documented examples. One is reported from southwestern Australian *Stylidium* (Stylidiaceae), where conspecific populations have diverged from one another such that they overlap less with sympatric congeners in location of stigma and anther contact with pollinators than would be expected by chance (Armbruster *et al.* 1994). A similar situation has been documented in Andean *Burmeistera* (Campanulaceae) by Muchhala and Potts (2007).

The fourth axis of potential ecotypic divergence is in flowering time. There is some evidence that ecotypes can diverge in the time of day or season that flowers are receptive for pollination. For example, pollination ecotypes of *D. scandens* tend to differ from each other in the time of day that their blossoms open, and this again follows a character-displacement pattern from sympatric congeners (although not statistically significant; Armbruster and Herzig 1984; Armbruster 1985). Interspecific variation in time of anthesis and pollen release in both African and neotropical *Acacia* sl. (Stone *et al.* 1998; Raine *et al.* 2007) suggests another system to investigate for ecotypic divergence. However, the large constraints (phylogenetic signal) on seasonal variation in flowering time (Kochmer and Handel 1986; Davies *et al.* 2013) suggest that ecotypic divergence in flowering phenology may be less common, although there are some good examples (see Anderson *et al.* 2010; Peter and Johnson 2014). Note that seasonal flowering-time divergence of species has a long history of study in the context of community assembly and species coexistence (e.g. Stiles 1975; see review in Rathcke and Lacey 1985).

### Divergence of species

Most insights into major shifts in pollination ecology come from comparing related species. Modern studies have based such comparisons on phylogenetic information, now mostly molecular phylogenies. The main issue considered is how and why shifts in pollination systems occur. To address this question, it is useful to differentiate the kinds of shifts that can occur. Stebbins (1974) argued that all shifts between pollination systems occurred during an intermediate phase, during which both old and new pollinators were effective. Stebbins' argument here is that, for natural selection to be important in switches between pollinators, there must be a series of variable intermediate phenotypes for it to act upon. If the old pollinator is lost immediately upon the new one being gained (resulting in reproductive isolation), then only ‘species selection’ (differential extinction and/or speciation of lineages) ‘chooses’ between the two new lineages (Stanley 1975; Gould 2002; Rabosky and McCune 2010; see also Fernández-Mazuecos *et al.* 2013).

Stebbins' gradualistic view has been countered by work on orchids (Dodson 1962; Dressler 1968; Schiestl and Ayasse 2002; Ayasse *et al.* 2011; Vereecken *et al.* 2011; but see Bradshaw *et al.* 2010), and more recently work on the molecular basis of pollinator discrimination (Schemske and Bradshaw 1999; Bradshaw and Schemske 2003; Yuan *et al.* 2013). These studies indicate that small genetic changes might sometimes lead to ‘instantaneous speciation’.

#### Studies of congeneric divergence

Most evidence for how shifts in pollination systems occur comes from study congeneric species. The number of such studies has grown tremendously in recent years and include Armbruster (1993; *Dalechampia*, Euphorbiaceae), Hapeman and Inoue (1997; *Platanthera*, Orchidaceae), Baum *et al.* (1998; *Adansonia*, Malvacae/Bombacaceae), Johnson *et al.* (1998; *Disa*, Orchidaceae), Kay *et al.* (2005; *Costus*, Costaceae), Perez *et al.* (2006; *Schizanthus*, Solanaceae) and Wilson *et al.* (2007; *Penstemon*, Plantaginaceae). Sometimes additional questions are addressed using data on the evolution of pollination systems. For example, Armbruster and Baldwin (1998) used *Dalechampia*, and Tripp and Manos (2008) used *Ruellia* (Acanthaceae) as comparative study systems for assessing whether or not specialization pollination can transition to generalized pollination (see also Martens-Rodriguez *et al.* 2010). Ley and Classen-Bockhoff (2009) commented on the role of intermediate forms in pollinator shifts in African Marantaceae. Smith *et al.* (2008), Martens-Rodriguez *et al.* (2010) and Sakai *et al.* (2013) examined the association between transitions in floral traits and functional groups of pollinators of flowers of *Iochroma* (Solanaceeae), Gesnerieae (Gesneriaceae) and Bornean gingers (Zingiberaceae), respectively, finding that some evolving floral traits were tightly associated with transitions between pollinators while others were not (see also Thomson and Wilson 2008). Friedman and Barrett (2008) conducted similar analyses of both order and direction of change of traits associated with shifts from animal to wind pollination across the angiosperms.

Very few studies have attempted to classify or tally the kinds of shifts (as described above) between pollination systems. Only two studies of which I am aware have tallied the frequencies of different types of pollinator shifts. The first is of *Dalechampia* (Armbruster 1993). Here 14 reconstructed shifts between pollinator systems were identified as quantitative, 3–6 as qualitative with the intermediate phase and 1–3 were identified as qualitative without the intermediate phase. The second study is of the Bignonieae (Bignoniaceae; Alcantara and Lohmann 2010), wherein the authors described 10 shifts with an intermediate phase and 19 without the intermediate phase (although this is based on floral morphology not pollinator observations). The first study largely supported Stebbins' gradualistic principle, but as a trend not a rule; i.e. most shifts had an intermediate stage during which natural selection could act to push or reverse the shift. Importantly, however, at least one shift appeared to lack any intermediate phase, and this could lead to instantaneous, non-adaptive speciation (by a single mutation affecting fragrance chemistry, leading to attraction of male euglossine bees instead of females). The second study suggested that shifts not involving the intermediate phase are much more common than Stebbins thought, although there is some uncertainty in this conclusion due to the lack of direct pollination data. The second study suggests an important role for non-adaptive speciation, at least in the Bignonieae. However, because all estimated phylogenies are incomplete representations of the true evolutionary history, these and other trends noted in this paper need to be assessed in many additional studies before placing too much confidence in any broad conclusions.

#### Increasing disparity without pollinator shifts

Not all increases in phenotypic disparity are associated with shifts in pollinators. For example, in *Dalechampia* over half (15) of 29 reconstructed speciation events associated with increased quantitative phenotypic disparity (morphological divergence) involved no change in pollinators (reanalysed from data in Armbruster 1993). Ellis and Anderson (2012) reviewed the topic recently and concluded that phenotypic divergence in the absence of pollinator change can come about in several ways. Behavioural variation in a single pollinator species can impose divergent selective pressures on plant species and drive adaptive divergence. Similarly, regional variation in the models that non-rewarding flowers mimic can generate disparity even though the same pollinator species is employed (Ellis and Anderson 2012). Variation in non-pollinator selective agents (e.g. florivores, seed predators) can also drive the evolution of disparity even when pollinators do not differ (e.g. Pérez-Barrales *et al.* 2013; see the section above on conflicting selection).

When related species share pollinators in sympatry, they may often diverge phenotypically, as, for example, the morphological diversity seen in *Pedicularis*, even though nearly all are pollinated by similar bumble bees (Macior 1983; Grant 1994*b*; Eaton *et al.* 2012; Huang and Shi 2013; Armbruster *et al.* 2014*b*). Similarly, floral disparity in *Stylidium* (Stylidiaceae) and *Burmeistera* (Campanulaceae) seems to be associated with divergence in sites of pollen placement on the same pollinator taxa instead of using different pollinator species (Armbruster *et al.* 1994, 2004, 2009*a*; Muchhala and Potts 2007).

## Speciation and Macroevolution

One of the major evolutionary discussions in the late 20th century was whether macroevolution was merely an extension of microevolution over longer time periods, or whether macroevolution was, to some extent, decoupled from microevolution (e.g. Gould 1980). Although the debate has died down, there has not really been any resolution, with paleobiologists still tending to favour decoupling and neontologists favouring lack of decoupling. If speciation is largely adaptive, then it forms a link between micro- and macroevolution and the two are coupled to some considerable extent. However, if speciation is largely non-adaptive, then micro- and macroevolution are largely decoupled (Stanley 1975; Gould 2002).

It is thus useful to understand more about how speciation occurs in flowering plants. Whenever flowering-plant speciation is accompanied by genetically based quantitative shifts in pollinators or by qualitative shifts in pollinators with an intermediate phase, then the speciation even is potentially adaptive, as in ecological speciation (Nosil 2012). Thus the relative frequency of instantaneous (non-adaptive) pollinator shifts, such as between sexually deceptive systems (e.g. floral volatiles mimicking insect sex pheromones) or between euglossine pollinators, relative to the other two forms gives us insights into the degree to which macroevolution might be decoupled from microevolution. The limited surveys to date suggest that both potentially adaptive (quantitative and qualitative with an intermediate phase) and potentially non-adaptive (without an intermediate phase) pollinator shifts occur, and the latter could lead to instantaneous speciation in some cases. However, overall, it appears that adaptive shifts are probably more common, suggesting potentially strong links between micro- and macroevolutionary patterns and processes. Note, however, that the potential role of natural selection in gradual shifts between pollinators does not, by itself, demonstrate its importance.

## Discussion

The rapid increase of species diversity (i.e. the increase of clade species richness through speciation) with increasing phenotypic disparity (phenotypic diversity) is usually termed an ‘adaptive radiation’ (Schluter 2000). The accumulation of disparity with species diversity may drive further adaptive speciation as a positive feedback. Any feature that increases the likelihood of reproductive isolation may thus have major effects on both diversification and phenotypic disparity. Features that can respond to selection for reinforcement (increased reproductive isolation as a result of selection on partially inter-fertile species in sympatry; but see Moyle *et al.* 2004) or selection for reproductive character displacement (selection against inter-mating in the absence of inter-fertility) thus may play a disproportionate role in ramping up clade species richness and phenotypic disparity (but see Rabosky and Matute 2013 for two animal counter-examples).

It is important to remember that there are strong fitness costs to interspecific pollination, even if species are completely inter-sterile and fully reproductively isolated (e.g. Muchhala *et al.* 2010). Further, unlike in reproductive isolation, only a small percentage improvement in getting pollen to, and from, the right species can be biologically significant and selected for. In contrast, for initial reproductive isolation or reinforcement, such small improvements would usually be genetically inconsequential.

It is also important to remember that most mechanical ‘isolation’ in plants is probably too incomplete to be of any biological significance in reproductive isolation (Armbruster and Muchhala 2009; Armbruster *et al.* 2014*b*), except in plants with pollinia, like orchids, or in combination with other isolating factors (Kay and Sargent 2009). However, placing pollen in different average locations on shared pollinators, even with overlap, is biologically significant because it enhances the reproductive fitness of individuals.

Adaptive inaccuracy and its components, maladaptive bias and adaptive imprecision, provide useful insights into the fitness costs and benefits of floral features, such as herkogamy (Armbruster *et al.* 2009*a*, *b*) and differences between sympatric species sharing pollinators (e.g. Armbruster *et al.* 2014*b*). In the few species that have been studied, low adaptive accuracies are generated primarily by low precision, especially in pollen placement (Armbruster and Muchhala 2009; Armbruster *et al.* 2009*a*, 2014*a*). Imprecision increases dramatically when accounting for realized inaccuracy, even in species with highly specialized flowers (Armbruster *et al.* 2014*b*). For example, large increases in imprecision (from fundamental to realized) have been observed in *Stylidium* (Stylidiaceae), where the increase is caused primarily by variation in pollinator orientation on landing on the flowers and/or pollen redistribution after the visit (W. S. Armbruster and J. A.Wege, unpubl. res.). These observations underscore the difficulty flowers with granular pollen have in achieving significant mechanical isolation by placing pollen in different locations on the same pollinators.

### Prospects for further research

**Figure 6.**
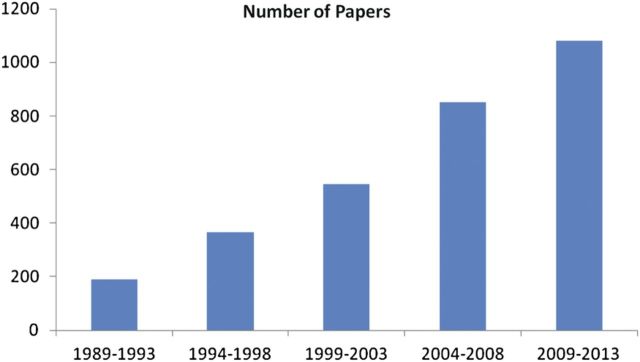
Number of papers on evolutionary aspects of pollination published in 5-year periods from 1989 to 2013. Data from Thomson ISI Web of Science, searching on “evolution AND pollination” on 30 Oct. 2013. Note that papers published prior to 1988 were excluded from the analysis because they did not include searchable abstracts.

Despite a long history of research, the evolution of floral specialization, plant–pollinator interactions and angiosperm diversity remain fruitful areas of investigation, indeed ones that have grown dramatically in recent years (Fig. 6). One reason that this area of research has experienced an explosion in activity in the past few decades, and a reason for optimism about the future vibrancy of the field, is that the interaction between plants and pollinators is a measurable phenomenon that is tightly linked to reproductive fitness. Thus both ecological and comparative studies have the capacity to address challenging, fundamental evolutionary questions, as well as questions that have practical applications in the conservation of biological diversity and the assessment of the impacts of global change on ecosystem services (e.g. pollination). Nevertheless, this review has probably identified more uncertainties than well-established knowledge.

A good understanding of fitness trade-offs in the interaction between floral features and pollinator effectiveness is critical to understanding the evolution of floral specialization. However, fitness trade-offs have been so rarely studied that we still do not know if they are common or uncommon. Especially rare are controlled experimental studies, which are the most unambiguous way to detect trade-offs. More studies using factorial experiments with different species of pollinators and different floral phenotypes would be valuable (see Wilson and Thomson 1996; Aigner 2001, 2006; Muchhala 2007). Another line of investigation not yet pursued is whether fitness trade-offs in pollination have influenced long-term clade success. Do lineages without fitness trade-offs have different diversification rates than those with trade-offs, e.g. because those without trade-offs have reduced extinction, or because those with trade-offs have higher speciation rates? It should therefore be a fruitful line of investigation to assess in well-studied groups the diversification consequences of the presence vs. absence of fitness trade-offs across lineages.

Much discussion of the role of pollinator constancy in the reproductive isolation of sympatric plant species has accumulated in the literature. Yet outside bumble and honey bees, we know very little about constancy in most pollinators. There are good reasons to think it may be facultative at most, and in many situations and organisms, including some bees, it may be low to non-existent (e.g. Armbruster and Herzig 1984; Raine and Chittka 2005; Pohl *et al.* 2011; Ellis and Johnson 2012; but see Goulson and Wright 1998). There is some evidence that floral complexity that increases the bee's learning time in achieving efficient handling promotes greater constancy (Corbet *et al.* unpublished; but see Wilson and Stine 1996). If so, this could contribute to higher speciation rates in complex flowers and hence explain greater clade diversity for lineages with complex or difficult-to-handle flowers (e.g. Sargent 2004; Kay and Sargent 2009).

To date there are very few phenotypic selection studies of ecotypes showing that contemporary selection maintains phenotypic differences between populations. This area deserves much more work. This is particularly challenging because year-to-year variation means that evolutionarily significant differences in selection may not be detected in any single year, even when they have been important in the phenotypic and genetic divergence of populations.

It remains a significant challenge to distinguish between the role of floral signals and morphology in generating or maintaining reproductive isolation vs. simply improving reproductive fitness in sympatry. The situation is not helped by the fact that ‘floral isolation’ is commonly used to refer to any degree of segregated pollen flow caused by flower or pollinator differences, even when it results in only a small percentage improvement. I fear that this has led some to think that reproductive isolation can commonly be generated by floral differences alone or in combination with other weak factors (‘components of reproductive isolation’). Given that only a tiny amount of inter-morph pollination will swamp any divergence except that driven by strong selection (Wright 1951; Roughgarden 1979; Waser 2001; Armbruster and Muchhala 2009), it is important to assess the degree of reproductive isolation that can result from different kinds of floral specialization. One critical feature is the fidelity of pollinator species and individuals visiting specialized flowers (ethological isolation; cf. Waser 1998, 2001; Chittka *et al.* 1999) and the degree to which pollen placement and stigma contact in different locations precludes inter-morph pollination (mechanical isolation; Grant 1994*a*). However, when pre-pollination isolation is incomplete, but no hybrids are observed in the field, as may often be the case (Cozzolino *et al.* 2005; Cozzolino and Scopece 2008; Armbruster and Muchhala 2009), one has to suspect that post-pollination isolation is what really matters in the origins of species. Pre-pollination differences would then more likely reflect adaptation to sympatry (reinforcement or character displacement). However, all this being said, we still cannot make generalizations because we lack broad enough surveys of the association between hybridization and pre- vs. post-pollination isolation (Armbruster and Muchhala 2009; cf. Rabosky and Matute 2013).

The relationship between pollinator shifts and speciation remains an exciting line of investigation (see van der Niet and Johnson 2012; Dell'Olivo and Kuhlemeier 2013; Nakazato *et al.* 2013). New methods of estimating changes in diversification and speciation rates in lineages hold much promise for assessing the influence of pollinator shifts on rates of diversification (Armbruster *et al.* 2009*b*; Smith 2010). It might also be interesting to assess the association between different kinds of shifts and diversification. One early classification described, as discussed above, three kinds of pollinator shifts (quantitative, qualitative with an intermediate phase and qualitative shifts without an intermediate phase). This classification may need refining and certainly needs much more empirical testing. At the moment, however, it still seems a reasonable way to approach the process because of its direct connection to the causes and evolutionary consequences of pollinator shifts, potentially yielding insights into whether pollinator shifts (and speciation) are largely adaptive vs. non-adaptive and informing the micro–macroevolution discussion.

Also at the macroevolutionary scale, it will be interesting to explore the role of key innovations in the evolution of phenotypic disparity. At present, key innovations are investigated by looking at their putative effects on diversification rates. But of equal interest is the effect of a trait on both total disparity and lineage-corrected disparity (i.e. the morphological or ecological ‘diversity’ corrected for the number of lineages or species in the clade). Another line of investigation that may prove promising is to examine the evolutionary lability vs. conservatism of pollinator associations in relation to attraction traits (rewards, advertisements) and traits affecting floral–pollinator fit and pollen/stigma contact points. Is there a stronger phylogenetic signal in some features than others? Are sister species more likely to differ in one of these and be the same in the others? Is the evolution of certain features (e.g. reward chemistry) more constrained than others, and if so why? Does the evolutionary change in reward chemistry associated with pollinator shifts generally precede or lag behind evolutionary change in other traits?

Are diversity and floral disparity correlated in a non-trivial manner (beyond the sampling effect), and if so, how (see Rabosky *et al.* 2013)? Does speciation promote phenotypic evolution (Eldredge and Gould 1972; Gould 2002), or does floral evolvability promote speciation (Bolstad *et al.* 2014; see also Rabosky *et al.* 2013)? Alternatively, might the evolvability of other aspects of phenotype (e.g. vegetative morphology, ion-uptake physiology) promote speciation, and increasing floral disparity reflect adaptive response to packing numerous sympatric species into the same ‘pollination space’ (see Armbruster *et al.* 1994; Armbruster and Muchhala 2009)?

## Sources of Funding

The research was supported by grants from the US National Science Foundation, the Norwegian Research Council, The British Council and the UK Royal Society.

## Conflicts of Interest Statement

None.
